# Case of Fungus Hæmatodes of the Stomach and Liver

**Published:** 1830-04-01

**Authors:** 


### SOUTH-EASTERN DISTRICT GENERAL DISPENSARY.

REMARKABLE CASE OF FUNGUS HÆMATODES IN THE STOMACH AND LIVER; WITH THE APPEARANCES ON DISSECTION. By J. THWAITES, M.D. Physician to the above-mentioned Dispensary.

November 17th, 1829. Eliza Maguire, æt. 50 years, has been affected for some months past with frequent attacks of pain in the stomach and abdomen, resembling colic, sometimes attended with vomiting of blood. Appetite very unequal—at intervals so craving, that nothing seemed to be too nauseous for her stomach, and exciting the *greatest impatience* for food, so much so that she would frequently (to use the expressions of her relatives) "snatch the meat from the vessel in which it was preparing, even before it was fit for use;" bowels generally costive; tongue foul, and an unpleasant taste continually in her mouth; skin cool and soft; strength tolerably good; countenance pale, but not much emaciated; no cough or dyspnœa; sleeps well; spirits very good; is not apprehensive of danger. She has three tumours in the epigastric region, each about the size of a small apple, perceptible to the view as well as to the touch—hard, apparently lobulated, as if composed of smaller tubercles, united into one large tumour. They are moveable to a certain extent, and not painful when subjected to tolerably strong pressure.

States that, some months ago, she passed two tape worms of considerable size; that her mother died of two tumours in the abdomen; that her brother nearly lost his life about two years ago from a severe attack of hæmatemesis, and that she lost a daughter from the same cause. Her husband has been dead many years—she has had four children.

Nov. 18th. Pulse natural; tongue white; bowels confined; no pain of abdomen on pressure; total disinclination for food; no thirst; spirits good.

℞. Pilul. Rhei c. grana x.
—— Hyd. gr. x.
Ox. Bismuthi, ℈j. M. ft. pilul no. viij. una ter de die sumenda.

19th. States that she feels somewhat better to-day; slept well last night; bowels affected by medicine twice; stools dark-coloured; anorexia continues; pulse natural; spirits very good. Lumps unaltered. Cont medicamenta.

21st. Continues to improve; tumours apparently softer, and one somewhat reduced in size; bowels moved regularly each day; pulse natural; tongue cleaner; appetite better; she is very anxious to have some broth, which was permitted. Cont. med.

℞. Hydriod. Potassæ, ℈ij.
Ung. Hyd. ℥ss.
Liq. Potass. gutt. x.
Ung. Simpl. ℥jss. M. ft. unguentum; cujus ʒj. super tumores illinetur, nocte maneq.

From this time until the 26th, she did not experience any material alteration in her symptoms. The tumours became softer—one altogether disappeared; the bowels were free, and the stools became each day more dark, as if mixed with grumous blood. On the 26th, she expressed considerable anxiety about the state of her stomach, which she described to be full and distended; bowels in the same state, tongue not so clean; not any perceptible change in the feel or appearance of the abdomen to pressure, except in the lumps, which appeared more moveable, and admitted the interposition of the fingers when much pressed. Omittr. medic.

I was sent for in great haste this evening about 7 o'clock, having been told that she was dying. When I entered her room, a great part of the floor was covered with blood and pus, as if discharged from an extensive abscess; a small bowl full of blood also lay beside her bed. She was considerably agitated—strength much reduced—pulse 130, small and weak—her voice far from strong, but her articulation quite distinct—abdomen and tumours unaltered—bowels had been freed a short time before the discharge from her stomach; but there was no unusual appearance in her stools.

Ordered a restorative draught of water of ammonia, tinct. rhei, and camphor mixture—also a solution of muriate of soda in water having been recommended by a friend, no objection was made, and she was directed to take a table-spoonful every two hours.

29th. She was much revived by the draught; no return of the vomiting since; pulse 120, stronger; bowels freed twice since—passed much blood.

Tongue furred and loaded; thirst considerable; total loss of appetite; tumours in the same state; not painful to pressure when not severe; spirits somewhat revived. Is apprehensive of a return of the hæmorrhage; countenance composed.

28th. Continues in the same state; no return of vomiting; bowels free; stools dark; pulse 120, small; tongue white; skin soft; spirits good; no pain. To resume the medicines.

29th. Complains of great fulness of the stomach, a frequent sensation of something rising in her throat, which she kept down by as frequently swallowing it again; bowels free; stools the same; tongue white; pulse 120; tumours in the same state. Continuantur medicam.

This evening she had a return of the hæmatemesis, from which she felt greatly exhausted; extremities cold; still free from pain. Endeavoured to restrain the flow of blood by swallowing it as it came to her mouth. About 10 o'clock a repetition of the hæmorrhage ensued, and in a violent exertion to discharge her stomach she expired.

*Sectio Cadaveris.* External characters:—Countenance calm; extremities not much emaciated; abdomen much swollen, but by no means tense; tumours concealed from the view by the abdominal distention, but very evident to the touch.

Upon cutting into the abdomen, the first appearance which presented itself was a large abscess on the internal surface of the right lobe of the liver, close to its anterior edge. It was filled with a white purulent fluid, resembling thick cream, not exhaling any peculiar odour. On the surface and in the substance of the liver were to be found numerous small tubercles, about the size of a nut, in various states—some hard and chalky, others softer, and many more in a state resembling that first described; the gall-bladder was thickened in its coats, white in its external appearance, and full of calculi of various sizes; adhesions were also evident between the liver and surrounding viscera. The spleen somewhat larger than usual. The duodenum and small intestines distended, and containing large quantities of coagulated blood. Mesenteric glands considerably enlarged, and of a pale or rather white appearance.

In the stomach, however, lay concealed the chief cause of her last illness. External to the pyloric orifice there was a small appearance of ulceration, which, however, did not penetrate the coats; the internal surface was slightly inflamed around the pylorus; the parts intervening between that and the cardiac orifice were perfectly sound, if we except the thickening of its coats. Immediately adjoining the cardiac orifice was a large tumour, ulcerated, and of a dark chocolate colour; the first view strikingly resembled fungus hæmatodes deeply ulcerated; its substance was the same as the tubercles before described, and when broken between the fingers, gave out a dark-coloured watery fluid. Around it were numerous smaller tumours, which gave a tuberculated and thickened feel to that part of the viscus. When washed, the tumour did not lose its dark appearance. The contents of the stomach chiefly consisted of thick clots of blood. The lungs were perfectly healthy, and the heart equally sound in appearance, but rather soft to the touch, or when cut into with the scalpel.

The brain was not examined.

I have been thus minute in the post-mortem description, as I consider that, in diseases of the stomach, the pathology of which is still veiled in such obscurity, whatever may, even in the most remote degree, tend to throw a light upon the nature of these diseases, must be valuable to the profession at large, and although the case here described may still further prove the inefficacy of medicine in *removing* the source of suffering, yet much may be learned as to the palliative treatment under similar circumstances in future, from the perusal of cases similar to the present. Had the extract of conium, hyosciamus, or of opium been employed instead of the purgative plan pursued, it is more than probable, that whatever might have been her sufferings, she still might have lived to partake of the sorrows or enjoyments of a world to which she had been wedded by an awful, and to her friends a melancholy, attachment.

N.B. This person never suffered from the jaundice, and she had no symptom of hepatic disease, any further than such as were connected with the state of her stomach.

